# Population Genetics of *Anopheles coluzzii* Immune Pathways and Genes

**DOI:** 10.1534/g3.114.014845

**Published:** 2014-12-30

**Authors:** Susan M. Rottschaefer, Jacob E. Crawford, Michelle M. Riehle, Wamdaogo M. Guelbeogo, Awa Gneme, N’Fale Sagnon, Kenneth D. Vernick, Brian P. Lazzaro

**Affiliations:** * Department of Entomology, Cornell University, Ithaca, New York 14853; † Department of Microbiology, University of Minnesota, St. Paul, Minnesota 55108; ‡ Centre National de Recherche et de Formation sur le Paludisme, 01 BP 2208 Ouagadougou, Burkina Faso; § Department of Integrative Biology, University of California, Berkeley, Berkeley, California 94720; ** Institut Pasteur, Unit of Insect Vector Genetics and Genomics, Department of Parasites and Insect Vectors, CNRS Unit of Hosts, Vectors and Pathogens (URA3012), Paris 75015, France

**Keywords:** population genetics, innate immunity, balancing selection, C-type lectin, JAK-STAT, genetics of immunity

## Abstract

Natural selection is expected to drive adaptive evolution in genes involved in host–pathogen interactions. In this study, we use molecular population genetic analyses to understand how natural selection operates on the immune system of *Anopheles coluzzii* (formerly *A. gambiae* “M form”). We analyzed patterns of intraspecific and interspecific genetic variation in 20 immune-related genes and 17 nonimmune genes from a wild population of *A. coluzzii* and asked if patterns of genetic variation in the immune genes are consistent with pathogen-driven selection shaping the evolution of defense. We found evidence of a balanced polymorphism in *CTLMA2*, which encodes a C-type lectin involved in regulation of the melanization response. The two *CTLMA2* haplotypes, which are distinguished by fixed amino acid differences near the predicted peptide cleavage site, are also segregating in the sister species *A. gambiae* (“S form”) and *A. arabiensis*. Comparison of the two haplotypes between species indicates that they were not shared among the species through introgression, but rather that they arose before the species divergence and have been adaptively maintained as a balanced polymorphism in all three species. We additionally found that *STAT-B*, a retroduplicate of *STAT-A*, shows strong evidence of adaptive evolution that is consistent with neofunctionalization after duplication. In contrast to the striking patterns of adaptive evolution observed in these *Anopheles*-specific immune genes, we found no evidence of adaptive evolution in the Toll and Imd innate immune pathways that are orthologously conserved throughout insects. Genes encoding the Imd pathway exhibit high rates of amino acid divergence between *Anopheles* species but also display elevated amino acid diversity that is consistent with relaxed purifying selection. These results indicate that adaptive coevolution between *A. coluzzii* and its pathogens is more likely to involve novel or lineage-specific molecular mechanisms than the canonical humoral immune pathways.

*Anopheles* mosquitoes live in pathogen-rich environments where survival requires an effective immune system. Population genetic studies in *Anopheles gambiae* have generally focused on putative antimalaria genes (Obbard *et al.* 2007, 2008, 2009a; Slotman *et al.* 2007; Cohuet *et al.* 2008; Parmakelis *et al.* 2008; White *et al.* 2011; Rottschaefer *et al.* 2011). However, mosquitos are also exposed to diverse microbes, microsporidia, and other pathogens during their larval stages when they live in septic standing water (Muspratt 1946; Bargielowski and Koella 2009; Wang *et al.* 2011), and these larval pathogens probably impose stronger selection on the immune system than the malaria parasite. Regardless of the proximal selective agent, any pathogen-driven evolution of the immune system is likely to shape the efficacy of resistance to parasites of public health relevance, including human malaria, through pleiotropic effects on cross-resistance (Mitri *et al.* 2009; White *et al.* 2011; Rottschaefer *et al.* 2011). In this study, we look broadly at the *Anopheles* immune system to determine targets of natural selection and identify potential instances of pathogen-driven evolution that may shape general resistance to infection.

Because host fitness depends on the ability to combat infection, pathogens are expected to impose significant selection pressure and immune system genes are often observed to evolve rapidly (Schlenke and Begun 2003; Nielsen *et al.* 2005; Obbard *et al.* 2006; Sackton *et al.* 2007; McTaggart *et al.* 2012). Comparative genomic studies show conserved gene orthology in insect innate immune systems, particularly for genes in intracellular signaling pathways, yet amino acid divergence between species is often elevated in these orthologs relative to nonimmune genes (Sackton *et al.* 2007; Waterhouse *et al.* 2007). Immunological divergence can additionally occur through lineage-specific and species-specific gene family expansions and contractions, which may reflect differences in selection pressures imposed by distinct pathogenic environments. This process is particularly pronounced in recognition and effector genes (Sackton *et al.* 2007; Waterhouse *et al.* 2007). These comparative genomic studies have provided insight into how the immune system evolves between species over longer evolutionary time, but they do not provide information about how natural selection operates to shape the immune system within a species over shorter timescales. Additionally, comparative genomic analyses are only able to reveal adaptive divergence and cannot detect the adaptive maintenance of polymorphism within species. Signatures of pathogen-driven evolution over shorter time scales can be detected more effectively from patterns of intraspecific polymorphism relative to divergence in immune system genes.

Insect immune systems can be broadly divided into categories of humoral and cellular responses. The humoral immune response involves recognition of pathogens by pattern recognition molecules that initiate intracellular signaling cascades that stimulate transcriptional activation of antimicrobial effector molecules. The Toll and Imd immune signaling pathways have been most extensively studied for their role in antibacterial and antifungal immunity in *Drosophila*, but these pathways have also been shown to play a role in immunity against a variety of pathogens in other species. In *A. gambiae*, the Imd pathway has been implicated in defense against both Gram-positive and Gram-negative bacteria (Meister *et al.* 2005, 2009; Garver *et al.* 2009) and has been shown to play an important role in mediating protection against the human malaria parasite *Plasmodium falciparum* (Mitri *et al.* 2009; Garver *et al.* 2009, 2012; Meister *et al.* 2009). The *A. gambiae* Toll pathway is involved in defense against bacteria and rodent malaria (Barillas-Mury *et al.*1996; Frolet *et al.* 2006, Riehle *et al.* 2008; Garver *et al.* 2009). The JAK-STAT pathway also plays a role in the response to bacterial infection in *A. gambiae* (Barillas-Mury *et al.* 1999; Gupta *et al.* 2009). Although most dipterans have only a single STAT gene, there are two STAT genes in *A. gambiae*, which appear to be the result of a retroduplication event along the Anopheline lineage. *STAT-A*, the ancestral gene, is most closely related to STAT genes in other insects, and *STAT-B* is a derived retrocopy (Gupta *et al.* 2009). The JAK-STAT pathway in *A. gambiae* is less well-studied than the Toll and Imd pathways, but studies indicate that it may play a role in killing *P. falciparum* parasites at the oocyst stage in *A. gambiae* (Gupta *et al.* 2009), and it has also been shown to limit *Plasmodium vivax* infection in *Anopheles aquasalis* (Bahia *et al.* 2011).

In the cellular immune response, pathogen recognition leads to encapsulation, phagocytosis, or melanization of the pathogen by hemocytes. The melanization response in particular has been studied in *A. gambiae* for its role in killing plasmodium parasites (Collins *et al.* 1986). Two C-type lectins, CTL4 and CTLMA2, have been shown to play a role in regulating the melanization response in *A. gambiae*. CTL4 and CTLMA2 primarily exist in the form of a heterodimer, which is secreted into the hemolymph (Schnitger *et al.* 2009). RNAi experiments indicate an important role for CTL4 and CTLMA2 in antibacterial immunity, because silencing of either gene reduces the number of Gram-negative bacteria melanized (Schnitger *et al.* 2009). In contrast, silencing of either CTL4 or CTLMA2 results in an increase in the number of melanized *Plasmodium berghei* ookinetes, suggesting that the CTL4/CTLMA2 heterodimer acts as an agonist for *P. berghei* parasites, preventing their melanization (Osta *et al.* 2004).

In this study, we examine patterns of genetic variation at 20 immune genes in a population of *Anopheles coluzzii* (formerly *A. gambiae* “M form”) (Coetzee *et al.* 2013) from Burkina Faso. The set of immune genes consists of genes belonging to the Toll, Imd, and JAK-STAT pathways, as well as other mosquito-specific immune factors. To control for demography and the effects of genomic location, we additionally sequenced 17 nonimmune control genes located physically nearby in the genome to our genes of interest. We found evidence that two distinct haplotypes in *CTLMA2* arose before the divergence of *A. coluzzii*, *A. gambiae* (“S form”), and *A. arabiensis*, and they have been adaptively maintained as a balanced polymorphism in all three species. We also found evidence of rapid adaptive evolution in *STAT-B*, suggesting an important role for the JAK-STAT pathway in *A. coluzzii*. We observe higher overall rates of amino acid divergence in the immune genes relative to the control genes. Genes involved in the Imd pathway show particularly high amino acid divergence, but they also display elevated amino acid diversity that is consistent with relaxed purifying selection.

## Materials and Methods

### Mosquito collection and DNA isolation

The *Anopheles coluzzii* individuals used in this study were collected in the village of Goundry, Burkina Faso (coordinates 12°30′N, 1°20′W) in September 2008. Freshly fed females were captured indoors by manual aspirator catch and DNA was extracted from individual carcasses using DNAzol (Invitrogen). Diagnostic PCRs were performed to confirm the species (Scott *et al.* 1993; Favia *et al.* 2001), and whole genome amplification of each sample was performed using the GenomiPhi V2 DNA Amplification Kit (GE Healthcare). *Anopheles merus* DNA from the OPHANSI colony was obtained from Malaria Research and Reference Reagent Resource Center (MR4).

### Loci analyzed

We sequenced a set of 20 immune genes including the Toll pathway genes *GNBPB1*, *TOLL1A*, *TUBE*, *PELLE*, *TRAF6*, *CACT*, and *REL1*, the Imd pathway genes *IMD*, *FADD*, *CASPL1*, *TAK1*, *IAP2*, *IKK1*, *IKK2*, and *REL2*, the JAK-STAT transcription factors *STAT-A* and *STAT-B*, and the *Anopheles* specific immune factors *CTL4*, *CTLMA2*, and *LRIM1*. To control for demography and background effects of genomic location, we additionally sequenced 17 nonimmune control genes located nearby our genes of interest. These controls are located within 40–100 KB of their "matched" immune genes and are similar in size and structure to the immune gene they are matched to. The names and relative chromosomal locations of all loci are shown in Supporting Information, Figure S1. All PCR primers were designed based on the published *A. gambiae* genome sequence (Vectorbase, *A. gambiae* genome, version P3). Loci were sequenced from a set of 20 *A. coluzzii* females. Some loci could not be amplified in all 20 individuals, but all loci were sequenced in a minimum of 18 *A. coluzzii* individuals as well as in *A. merus*.

### PCR and sequencing

Each gene was amplified in a single amplicon from whole genome amplified DNA using iProof high-fidelity DNA Polymerase (BioRad). PCR products were run out on a 1% agarose gel and the product fragments were excised and purified using EZNA gel extraction kits (Omega BioTek). Adenosine tails were added to the purified products by incubating for 20 min at 72° with PCR buffer, dATP, and Taq polymerase. Products were then cloned using either TOPO or TOPO XL cloning kits (both from Invitrogen). Colonies to be sequenced were grown overnight at 37° in liquid Luria-Bertani broth supplemented with 20 mg/ml kanamycin, and the plasmids were isolated using the Qiaprep spin miniprep kit (Qiagen). The products were sequenced directly from the plasmids using the BigDye Terminator Cycle Sequencing Kit v3.1(ABI). The sequences were assembled using Sequencher (Gene Codes Corp). All sequences have been deposited in GenBank under accession numbers KP274100-KP274844.

Only one of the two alleles at each gene was sequenced from any given mosquito in the study. To correct sequencing errors, all singleton polymorphisms were verified by re-amplification and direct sequencing of heterozygous PCR products. For singleton validation, the entire gene was amplified directly from the whole genome amplified DNA using iProof high-fidelity DNA Polymerase (BioRad), and this full-length amplicon was then used as a template for a secondary PCR that used internally nested primers to robustly amplify the gene region containing the singleton to be validated. Unincorporated primers and dNTPs were inactivated from these secondary amplification products by incubation with ExoI and SAP (both manufactured by USB), and amplification products were sequenced using the BigDye Terminator Cycle Sequencing Kit v3.1 (ABI).

### Population genetic analysis

Average pairwise genetic diversity (π) was calculated for all sites, and also separately for synonymous (π_s_) and nonsynonymous (π_a_) sites using DnaSP v.5 (Librado and Rozas 2009). The Tajima’s *D* statistic (Tajima 1989) was also calculated in DnaSP using silent sites only. The average pairwise genetic divergence at synonymous (K_S_) and nonsynonymous (K_A_) sites and their ratio (K_A_/K_S_) were calculated in DnaSP using *A. merus* as an outgroup. Maximum likelihoood multi-locus HKA tests were implemented using the mlhka program (Wright and Charlesworth 2004) using synonymous sites only. Multi-locus McDonald Kreitman tests were performed using the software MKtest v2.0 (Welch 2006; Obbard *et al.* 2009a). The multi-locus McDonald Kreitmen tests were performed on the full dataset, as well as on the Toll and Imd pathway genes separately. Three models, which varied only in the parameter α, were implemented for each dataset. In the first model (M0), α was fixed at zero for all loci. In the second model (M1), a single α value estimated from the data were shared by all loci, and in the third model (M2) α was estimated separately for the immune and control loci. For all three models, the expected neutral divergence (λ=µ*t*) and neutral diversity (θ=4*N_e_*µ) each took a single value at all loci, and selective constraint was allowed to vary between loci. Maximum likelihood ratio tests and Akaike weighting were used to assess model fit.

To identify distinct haplotype clades in CTLMA2, neighbor-joining gene trees were constructed in MEGA5 (Tamura *et al.* 2011) using the maximum composite likelihood method and uniform substitution rates, with 1000 bootstrap replicates. To determine if the CTLMA2 haplotype clades were present in other species, we compared our data with published CTLMA2 sequences of *A. gambiae* (GenBank accession numbers EF519453–EF519450 and EF519463–EF519478) and *A. arabiensis* (GenBank accession numbers EF519419–EF519428), both collected from Kenyan populations, as well as the outgroup species *A. quadriannulatus* (GenBank accession number EF519435) (Obbard *et al.* 2007). To test for introgression of the CTLMA2 haplotypes among *A. coluzzii*, *A. gambiae*, and *A. arabiensis*, we calculated the average number of pairwise differences (*D_xy_*) within and between each clade in DnaSP. To determine typical values of *D_xy_* in the genomic region of CTLMA2, we calculated *coluzzii-arabiensis D_xy_* and *gambiae-arabiensis D_xy_* from published data for the nearby loci AGAP005540 (GenBank accession numbers EF519480–EF519501) and APL2 (GenBank accession numbers EF519504–EF519528) (Obbard *et al.* 2007).

No position-matched control gene was sequenced for one immune locus (*TRAF6*), and in two instances a pair of immune loci were located very near to each other (*TAK1* and *PELLE* on chromosome 2R, *CTL4* and *CTLMA2* on chromosome 2L), so a single position control was sequenced for each pair (see Figure S1). Differences in divergence between the immune and control groups were assessed using Mann-Whitney *U* tests. When analyzing the full dataset, each control gene was included only once, but because *TAK1* and *PELLE* are involved in different signaling pathways (Imd and Toll, respectively), the shared control gene was included in the control group for analysis of each individual pathway. Differences in nucleotide polymorphism between the immune and control groups were assessed using paired Wilcoxon tests. *TRAF6*, which lacks a position control, was excluded from these comparisons, and the shared control genes were included twice as they were paired to each immune locus. Mann Whitney *U* tests and paired Wilcox tests for differences in divergence and diversity were implemented in R (R Development Core Team 2011).

## Results

### Reduced purifying selection in the IMD pathway

To test the hypothesis that *A. coluzzii* immune genes might evolve under positive selection, we analyzed patterns of intraspecific and interspecific genetic variation in 20 immune genes and 17 control genes from a single population in Burkina Faso. Population genetic statistics for each locus are listed in Table S1. Across all 37 genes, the average per-site nucleotide diversity was 1.3% at all sites (π), 2.6% at synonymous sites (π_s_), and 0.3% at nonsynonymous sites (π_a_). Nucleotide diversity was lower for genes on the X chromosome compared with the autosomes (X mean: π = 0.4%, π_s_ = 0.7%, π_a_ = 0.1%; autosome mean: π = 1.5%, π_s_ = 2.9%, π_a_ = 0.4%), in keeping with the lower effective population size on the X (Cohuet *et al.* 2008). Nucleotide divergence was measured using *A*. *merus* as an outgroup. The average per-site nucleotide divergence was 3.3% at all sites (K), 6% at synonymous sites (K_S_), and 0.9% at nonsynonymous sites (K_A_). These estimates for diversity and divergence are comparable with previously published estimates in *A*. *coluzzii* (Cohuet *et al.* 2008).

Genes that are the target of recurrent positive selection are expected to have elevated rates of amino acid evolution, which can be measured using the ratio of nonsynonymous to synonymous divergence (K_A_/K_S_). The average K_A_/K_S_ ratio of the immunity genes is significantly higher than that of the position-matched control genes (immune K_A_/K_S_ = 0.175; control K_A_/K_S_ = 0.077; Mann-Whitney *U* test *P* = 0.001) (Table 1). This difference is driven by divergence at nonsynonymous sites (K_A_), which is three-times higher in the immune genes than controls, whereas divergence at synonymous sites (K_S_) is not significantly different between the immune and control groups (Table 1). When we looked at genes involved in the Toll and Imd pathways separately, we found that genes involved in the Imd pathway have higher average K_A_/K_S_ values than their controls (Imd immune K_A_/K_S_ = 0.197; Imd control K_A_/K_S_ = 0.068; Mann-Whitney *U*-test *P* = 0.003) (Table 1). This difference is not driven by one or a few outliers, because the difference remains significant even after removing the highest three K_A_/K_S_ ratios. The average K_A_/K_S_ value in Toll pathway genes is also higher than their controls, although the difference is not statistically significant (Toll immune K_A_/K_S_ = 0.109; Toll control K_A_/K_S_ = 0.056; Mann-Whitney *U*-test *P* = 0.063) (Table 1). In both the Toll and Imd pathways, divergence at synonymous sites is not different between the immune and control groups, so the higher K_A_/K_S_ ratio in immune genes is attributable to elevated replacement divergence in the immune genes (Table 1). When all genes in the Imd pathway are excluded from the analysis, immune genes still have marginally higher average K_A_/K_S_ relative to the control genes (0.16 *vs.* 0.084; Mann-Whitney *U*-test *P* = 0.048) (Table 1), indicating that the Imd pathway is not the only driver of high amino acid divergence found in the immune genes.

**Table 1:** Average pairwise divergence in immune and control groups

	n*^a^*	K_total_*^b^*	K_S_*^c^*	K_A_*^d^*	K_A_/K_S_*^e^*
All data					
Immune	20	0.0335	0.0652	0.0120	0.1747
Control	17	0.0319	0.0545	0.0043	0.0772
		*U* = 149, *P* = 0.537	*U* =117.5, *P* = 0.113	*U* = 64.5, *P* = 0.001	*U* = 62.5, *P* = 0.001
IMD pathway					
Immune	8	0.0336	0.0613	0.0121	0.1968
Control	8	0.0302	0.0526	0.0041	0.0675
		*U* = 24, *P* = 0.442	*U* = 21, *P* = 0.279	*U* = 8, *P* = 0.010	*U* = 5, *P* = 0.003
TOLL pathway					
Immune	7	0.0332	0.0713	0.0087	0.1090
Control	6	0.0335	0.0565	0.0032	0.0555
		*U* = 22, *P* = 0.945	*U* = 14, *P* = 0.366	*U* = 8, *P* = 0.073	*U* = 7.5, *P* = 0.063
Exclude IMD pathway					
Immune	12	0.0335	0.0679	0.0119	0.1600
Control	10	0.0322	0.0557	0.0043	0.0835
		*U* = 58, *P* = 0.923	*U* = 44, *P* = 0.314	*U* = 28.5, *P* = 0.041	*U* = 29.5, *P* = 0.048

a Number of genes considered.

b Average per-gene divergence at all sites between *A. coluzzii* and *A. merus*

c Average per-gene silent divergence between *A. coluzzii* and *A. merus*

d Average per-gene replacement divergence between *A. coluzzii* and *A. merus*

e Average per-gene K_A_/K_S_ ratio between *A. coluzzii* and *A. merus.*

If the increased rates of amino acid evolution in the immune genes were due to recurrent fixation of beneficial alleles, then we would also expect to see reduced diversity at physically linked sites and a shift in the allele frequency spectrum (Nielsen *et al.* 2005). We found that diversity at synonymous sites was similar in the immune and control genes overall (immune π_s_ = 2.83%; control π_s_ = 2.52%; paired Wilcox V = 128; *P* = 0.196) (Table 3), but elevated in Imd pathway genes relative their controls (Imd immune π_s_ = 3.85%; Imd control π_s_ = 2.97%; paired Wilcox V = 34; *P* = 0.023) (Table 3). We found no difference in Tajima’s *D*, which measures shifts in the allele frequency spectrum, between the immune and control groups overall or when split by immune pathway (Table 3). We did, however, see significantly higher average polymorphisms at nonsynonymous sites in the immune genes relative to controls (immune π_a_ = 0.48%; control π_a_ =0.16%; paired Wilcox V = 172; *P* = 0.001) (Table 2), driven primarily by elevated π_a_ in the Imd pathway (Imd immune π_a_ = 0.66%; control π_a_ =0.18%; paired Wilcox V = 36; *P* = 0.008) (Table 3). The Toll genes do not have significantly higher average π_a_ than their controls (Toll immune π_a_ = 0.31%; control π_a_ = 0.14%; paired Wilcox V = 19; *P* = 0.219) (Table 3).

**Table 2:** Multilocus MK-test model comparison

Model	Description	Par	log(*L*)	2∆log(*L*)	χ2 *P* value	AICc*^a^*	Akaike Weight*^b^*	α_a_*^c^*	α_b_*^d^*
All loci									
M0	α = 0	38	−615.83			1335.9	0.806	[0]	[0]
M1	α ∼ (all loci)	39	−615.51	0.64	0.4237	1339.0	0.169	[0.07]	[0.07]
M2	α ∼ (control, immune)	40	−615.48	0.06	0.8065	1342.8	0.025	0.04	0.09
Imd Pathway									
M0	α = 0	18	−235.44			522.1	0.003	[0]	[0]
M1	α ∼ (all loci)	19	−228.01	14.86	0.0001	511.3	0.695	[−0.64]	[−0.64]
M2	α ∼ (control, immune)	20	−226.72	2.58	0.1069	513.0	0.302	−0.20	−0.91
Toll pathway									
M0	α = 0	14	−181.22			403.2	0.305	[0]	[0]
M1	α ∼ (all loci)	15	−178.55	5.34	0.0208	402.1	0.520	[−0.5]	[−0.5]
M2	α ∼ (control, immune)	16	−177.37	2.36	0.1245	404.3	0.175	−1.09	−0.24

a The Akaike information criterion corrected for sample size.

b The likelihood of the model, given the relative support for each of the models tested.

c Estimate of the proportion of adaptive substitutions in the control genes. Square brackets indicate where α is constrained by the model.

d Estimate of the proportion of adaptive substitutions in the immune genes. Square brackets indicate where α is constrained by the model.

**Table 3:** Average pairwise genetic diversity in immune and control groups

	n*^a^*	π_total_*^b^*	π_s_*^c^*	π_a_*^d^*	*D**^e^*
All data					
Immune	19	0.0140	0.0283	0.0048	−0.434
Control	19	0.0127	0.0252	0.0016	−0.764
		V = 102, *P* = 0.798	V = 128, *P* = 0.196	V = 172, *P* = 0.001	V = 141, *P* = 0.066
IMD pathway					
Immune	8	0.0182	0.0385	0.0066	−0.512
Control	8	0.0134	0.0297	0.0018	−0.601
		V = 27, *P* = 0.25	V = 34, *P* = 0.023	V = 36, *P* = 0.008	V = 20, *P* = 0.844
TOLL pathway					
Immune	6	0.0116	0.0231	0.0031	−0.353
Control	6	0.0141	0.0239	0.0014	−0.834
		V = 4, *P* = 0.219	V = 9, *P* = 0.844	V = 17, *P* = 0.219	V = 19, *P* = 0.094

a Number of genes considered, 18–20 alleles sampled per gene.

b Average per-gene genetic diversity calculated for all sites.

c Average per-gene genetic diversity calculated for synonymous sites.

d Average per-gene genetic diversity calculated for nonsynonymous sites.

e Average per-gene Tajima’s *D* calculated using silent sites.

The elevated Ka/Ks observed in immune genes could be consistent with positive selection driving adaptive evolution in these genes, but could also arise if immune genes are generally less constrained and therefore free to accumulate amino acid substitutions. Under a model of neutral evolution, the ratio of nonsynonymous to synonymous divergence is expected to equal the ratio of nonsynonymous to synonymous polymorphism. The McDonald-Kreitman test compares the number of polymorphisms and fixed differences at synonymous and nonsynonymous sites to detect deviation from the neutral expectation (McDonald and Kreitman 1991) and can be used to estimate the proportion of nonsynonymous fixations attributed to positive selection (α) (Smith and Eyre-Walker 2002). To determine whether the immune genes as a class show a higher proportion of adaptive substitutions than the control genes, we implemented a multi-locus MK test using the software MKtest v2.0 (Welch 2006; Obbard *et al.* 2009a). We first implemented the test on the full dataset. Using a likelihood ratio test, the fit of a model allowing α to take the maximum likelihood value showed no significant improvement over the null model where α is fixed at zero [M1 *vs.* M0 2∆log(*L*)=0.64; *P* = 0.4] (Table 2). We then implemented the test on genes in the Toll and Imd pathways separately. In both cases, the model that allowed a single value of α estimated from the data showed a significant improvement over the null model where α is fixed at zero [Imd M1 *vs.* M0 2∆log(*L*)=14.8, *P* = 0.0001; Toll M1 *vs.* M0 2∆log(*L*)=5.34, *P* = 0.0208] (Table 2). A model that allowed a separate α value to be estimated for the immune and control genes did not provide any additional improvement in model fit for either pathway [Imd M2 *vs.* M1 2∆log(*L*)=2.6, *P* = 0.1069; Toll M2 *vs.* M1 2∆log(*L*)=2.36, *P* = 0.1245] (Table 2). We thus see a proportional increase in both nonsynonymous polymorphism and nonsynonymous divergence in immune genes, providing no support for the hypothesis that the immune genes experience more positive selection than the control genes.

The elevated nucleotide diversity at both synonymous and nonsynonymous sites in the Imd pathway genes could be consistent with adaptive maintenance of polymorphism, or alternatively could indicate that purifying selection on these genes is weak relative to control genes, allowing deleterious nonsynonymous mutations to persist in the population as effectively neutral polymorphisms. The ratio of polymorphism to divergence is predicted to be equivalent across neutrally evolving loci, whereas elevated polymorphism relative to divergence is predicted under adaptive maintenance of polymorphisms. The HKA test (Hudson *et al.* 1987), which compares the ratio of polymorphism relative to divergence across multiple loci, can help distinguish between these hypotheses. We used a multilocus HKA test in a maximum-likelihood framework to compare the polymorphism to divergence ratios of the eight genes in Imd pathway genes with the other 29 genes in the dataset. Under this framework, a model allowing selection on Imd genes as a class did not show a significant improvement over a model that assumed all genes evolve neutrally [χ^2^_(8)_=6.52; *P* = 0.6]. These results indicate the elevated amino acid diversity observed in Imd pathway genes cannot be explained by a model of adaptive maintenance of polymorphism, but rather that these genes experience weakened purifying selection that allows deleterious nonsynonymous mutations to persist in the population as effectively neutral polymorphisms that may drift to fixation and contribute to divergence between species.

### Adaptive evolution in *STAT-B*

*STAT-B* is an intronless duplicate of *STAT-A* that arose through retrotransposition of a *STAT-A* mRNA. The duplication event occurred more recently than the divergence of the Anopheline and Culicine lineages (145-200MYA; Krzywinski 2006), yet the two STAT copies are remarkably divergent. STAT-A shows only 43% amino acid identity with STAT-B, whereas it retains 74% identity with *Aedes aegypti* STAT and 63% identity with *Culex quinquefasciatus* STAT. Despite the rapid divergence of *STAT-B*, there is no evidence of pseudogenization, and both STAT-A and STAT-B appear to play a role in immunity. STAT-A and STAT-B are differentially expressed in various developmental stages, with STAT-A being absent from the pupal stage when STAT-B is highly expressed and STAT-A being expressed at higher levels than STAT-B in adults (Gupta *et al.* 2009). Both STAT-A and STAT-B appear to be involved in resistance to bacteria and *Plasmodium* parasites (Gupta *et al.* 2009). Gene duplicates are often maintained in the genome because they acquire a function that is distinct from that of the ancestral gene. In this case, we might predict evidence of adaptive evolution in *STAT-B*, particularly when compared with *STAT-A*.

Synonymous site divergence between *A. coluzzii* and *A. merus* in *STAT-B* is typical of genes in our dataset (*STAT-B* K_S_ = 6.3%; mean K_S_ = 6%). Amino acid divergence, however, is remarkably high at this locus (*STAT-B* K_A_= 3%; mean K_A_= 0.9%), giving it the highest K_A_/K_S_ ratio in the dataset (*STAT-B* K_A_/K_S_= 0.46; mean K_A_/K_S_= 0.13). Such an excess of replacement divergence, along with a deficit of intraspecific nucleotide diversity (*STAT-B* π = 0.18%; mean π = 1.3%), is consistent with a model of recent adaptive evolution at this locus. To test the hypothesis that positive selection is driving the rapid divergence of *STAT-B*, we used the maximum likelihood multi-locus HKA test to test for a departure from neutrality in *STAT-B* as compared with all other genes in the dataset. A model that hypothesized that *STAT-B* was evolving by directional selection fit the empirical data significantly better than the null model that assumed neutral evolution in all genes [χ^2^_(1)_ = 9.1; *P* = 0.0026]. In contrast to the high amino acid divergence in *STAT-B*, *STAT-A* exhibits a complete lack of replacement divergence or polymorphism, consistent with a model of purifying selection. Estimates of total and synonymous polymorphism are low in *STAT-A*, but they are consistent with the estimates from other genes on the X chromosome. (X mean π = 0.42%, π_S_ = 0.7%; *STAT-A* π = 0.42%, π_S_ = 0.64%). This pattern of purifying selection in *STAT-A*, the ancestral gene, and rapid evolution in the derived duplicate *STAT-B*, is consistent with a model of neofunctionalization of *STAT-B*.

### Balancing selection in *CTLMA2*

**Figure 1:**
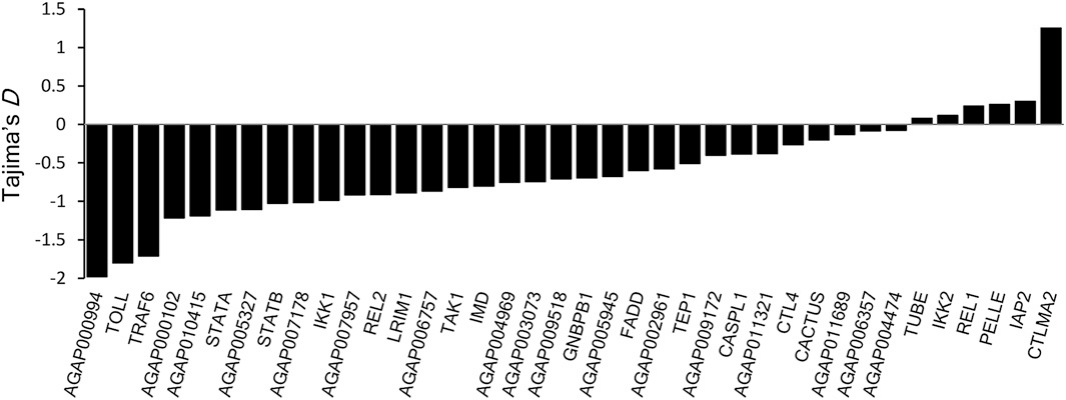
Distribution of Tajima’s *D* across all 37 genes. Tajima’s *D* was calculated for each gene using silent sites and plotted as a histogram showing deviation from neutral expectations. Loci are ordered based on the value of *D* to draw attention to the contrast between CTLMA2 and the rest of the dataset.

**Figure 2:**
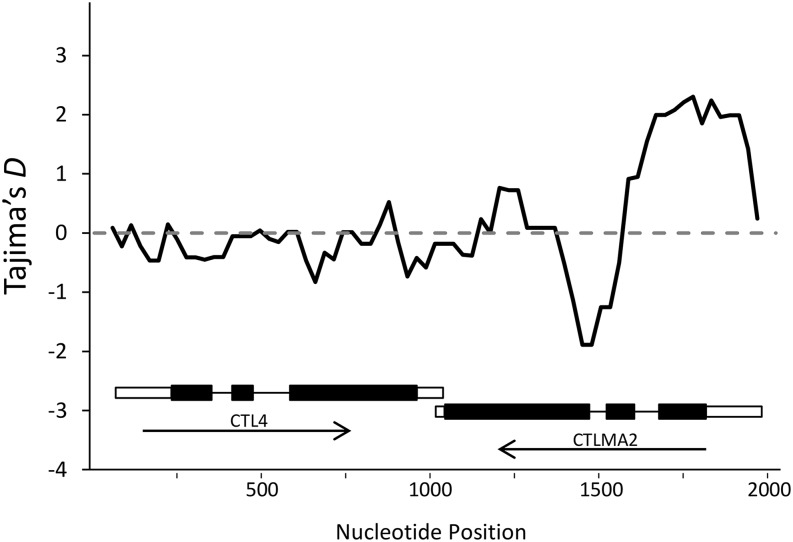
Sliding window analysis of Tajima’s *D* in CTL4 and CTLMA2. CTL4 and CTLMA2 are located directly adjacent on the chromosome and were PCR amplified and cloned as a single fragment. Tajima’s *D* was calculated using silent sites in a sliding window along the entire sequenced region using a 200-bp window with a 25-bp step size. The dashed line indicates the expected value of *D* under a neutral equilibrium model. The schematic below the plot shows the exon structure and direction of transcription for CTL4 and CTLMA2. Positive values of *D* in the 5′ region of CTLMA2 indicate an excess of intermediate frequency polymorphism in this region.

**Figure 3:**
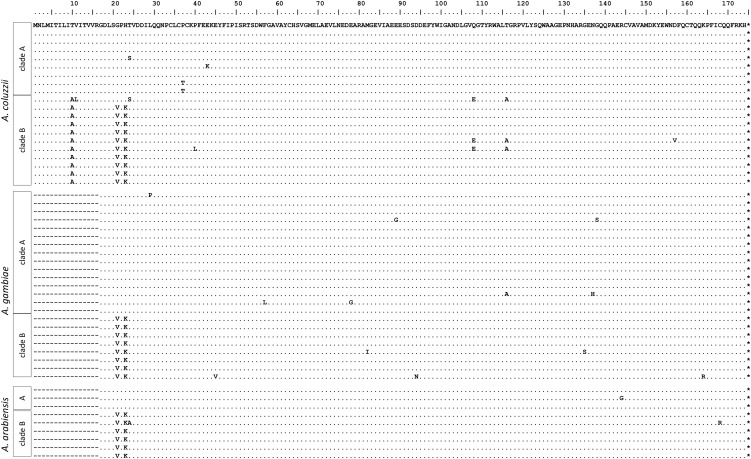
Amino acid alignment of CTLMA2 sequences of *A. coluzzii* collected in Burkina Faso, along with previously published sequences from *A. gambiae* and *A. arabiensis* collected in Kenya (Obbard *et al.* 2007). Sites matching the top sequence are indicated with a dot (•). Dashes (–) note unavailable sequence because we sequenced a different amplicon than was available for *A. gambiae* or *A. arabiensis*

CTL4 and CTLMA2 play a role in regulating the melanization response in *A. gambiae* (Osta *et al.* 2004). They primarily exist in the form of a heterodimer, which is secreted into the hemolymph (Schnitger *et al.* 2009). *CTL4* and *CTLMA2* are located directly adjacent on the chromosome and, in our study, were PCR amplified and cloned as a single fragment. Although all but three of the genes examined in this dataset have Tajima’s *D* values that are negative or nearly zero, *CTLMA2* stands out with a Tajima’s *D* value of positive 1.25 (Figure 1). A sliding window analysis of the entire *CTL4*/*CTLMA2* region shows that the high positive Tajima’s *D* values are limited to the 5′ end of *CTLMA2*, and that Tajima’s *D* throughout the rest of the gene is similar to that of *CTL4* (Figure 2). Inspection of the sequences reveals the presence of two distinct haplotype clades, hereafter referred to as clades A and B. There are 11 fixed SNP differences between clades A and B; all are located in the 5′ 350 bp of the gene. This block of fixed differences, which spans part of the 5′ UTR, the first exon, and the first intron, includes four SNPs that result in three amino acid changes near the predicted signal cleavage site (Figure 3). We considered that the divergent haplotype could have been introduced through paralogous gene conversion, but we were unable to find significant sequence matches to any CTL paralogs in either the *A. gambiae* genome or the entire NCBI nr database.

To determine if the presence of the two CTLMA2 haplotypes could be consistent with a partial selective sweep, a sweep with recombination, or a sweep from standing variation, we compared the patterns of genetic variation within each haplotype clade to genetic variation in *CTL4* and AGAP005327. If the presence of the haplotypes were due to a partial selective sweep, where the frequency of a selected allele increases in the population but does not or has not yet become fixed in the population, then we would expect to see evidence of selection in one clade, whereas variation in the other clade should be consistent with surrounding regions (Hudson *et al.* 1997). If the haplotypes are the result of a sweep from standing variation or a sweep with recombination, then we would expect both clades to show signatures of selection (Pritchard *et al.* 2010). It is important to note that the expected values of neutrality statistics like those used here are not the same for both a random sample of chromosomes and a partitioned subset of chromosomes. Most notably, the effective population size, which is a central parameter in these statistics, will be smaller in clade-wise estimates, because we are specifically conditioning on a specific subset of chromosomes in the population. In our sample, the clades are segregating at 50%, so we should expect an approximately 50% reduction in within-clade nucleotide diversity under neutral equilibrium conditions, assuming these haplotypes have segregated at this frequency for many generations. Tajima’s *D* is calculated as the normalized difference between two neutrality statistics that both depend on effective population size (Tajima 1989), so we do not expect the reduction in effective population size to affect this statistic. We found that estimates of nucleotide diversity and the site frequency spectra (Tajima’s *D*) of clade A are consistent with those observed in the adjacent *CTL4*, as well as the position-matched control gene AGAP005327 (Table 4). Nucleotide diversity is slightly lower in clade B, which also shows a nonsignificant shift in the site frequency spectrum, but on the whole there is little evidence of positive selection on either clade, so we cannot attribute the presence of the two CTLMA2 haplotype clades to partial or soft selective sweep.

**Table 4:** Population genetic statistics for CTLMA2 haplotype clades and surrounding loci

	n*^a^*	S*^b^*	π*^c^*	D*^d^*
CTLMA2 all	20	55	0.022	1.254
Clade A	10	30	0.010	−0.465
Clade B	10	25	0.007	−1.737
CTL4	20	44	0.013	−0.263
AGAP005327	19	54	0.010	−1.107

a Number of alleles sampled.

b Number of segregating sites.

c Average number of pairwise differences per site.

d Tajima’s *D* calculated using silent sites.

**Figure 4:**
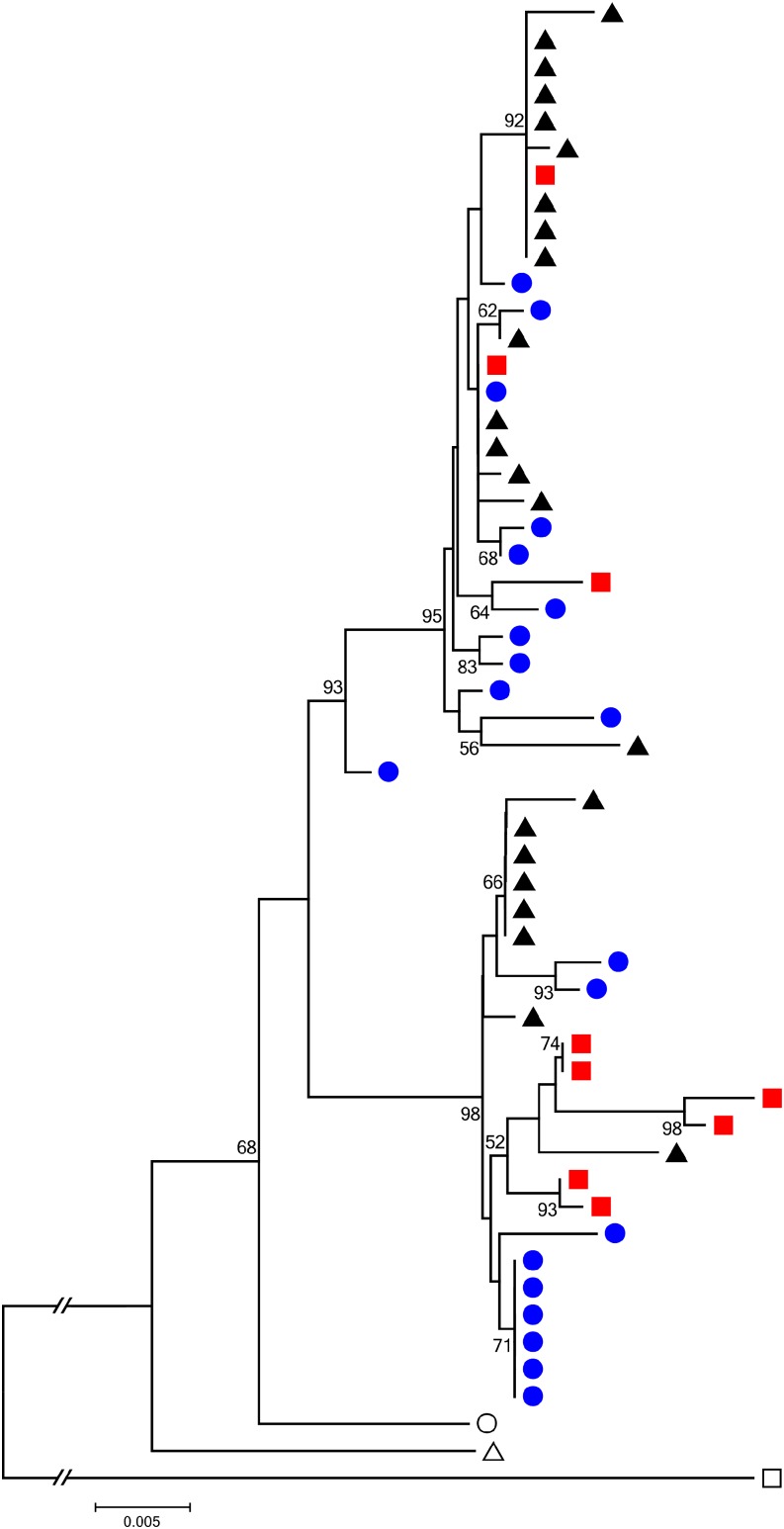
Neighbor-joining tree of *CTLMA2* sequences from *A. coluzzii* (blue circles), *A. gambiae* (black triangle), *A. arabiensis* (red squares), *A. quadriannulatus* (open circle), *A. merus* (open triangle), and *A. christyi* (open square). Bootstrap support was determined using 1000 bootstrap replicates, and nodes with >50% bootstrap support are shown.

Alternatively, these two divergent haplotypes may be balanced polymorphisms maintained by frequency-dependent selection, spatially varying selection pressures, or overdominance. We might then predict that these haplotypes have been segregating for evolutionarily long times and thus might also be found in closely related species. We tested this hypothesis by comparing our sequences to a published dataset (Obbard *et al.* 2007) and found that both CTLMA2 haplotypes are also segregating in *A. gambiae* (“S form”) as well as in *A. arabiensis* (Figure 3, Figure 4). The presence of both haplotypes in all three species could be consistent with an origin that predates the divergence of the species or, alternatively, they could have been shared among species through adaptive introgression. Introgressed loci are expected to be less diverged between species compared with adjacent chromosomal regions. Therefore, if the CTLMA2 haplotypes arose after the species diverged and were shared by introgression, then we would expect the introgressed haplotype to show relatively low divergence between species and divergence in the ancestral haplotype to be typical for the genomic region. If the CTLMA2 haplotypes predate the divergence of the species, then divergence within each haplotype clade should be similar to the genome average, whereas divergence between haplotype clades should be elevated, reflecting a longer coalescence time. To determine which of these models best fits the data for CTLMA2, we calculated the average number of pairwise differences, *D_xy_*, among species within and between the CTLMA2 haplotype clades, and also in two nearby loci from a published dataset (Obbard *et al.* 2007). Because *D_xy_* depends on effective population size (Nei 1987), which is lower within clades as noted above, we would expect D*_xy_* to be smaller relative to nearby regions without similar long-term haplotype structure. We found that *D_xy_* among species ranged from 0.008 to 0.012 within each haplotype clade (Table 5), and from 0.030 to 0.035 between haplotype clades (Table 5). The within-clade values are consistent with *D_xy_* among species calculated for two nearby loci (*D_xy_* = 0.016–0.018, Table 5) and estimated genome-wide *gambiae-arabiensis D_xy_* of 0.011 (O’Loughlin *et al.* 2014), whereas the between-clade values are approximately three-times higher, and even higher than the estimated genome-wide *gambiae-merus* D*_xy_* of 0.0235, most likely reflecting an ancient origin. Although we can only compare our values with the mean and SD among individual locus values of *D_xy_* in the O’Loughlin *et al.* (2014) dataset, our between-clade values are greater than the mean plus 1 SD of even the *gambiae-merus* comparison, suggesting that the CTLMA2 values exceed most of the many independent genealogies in their study. Moreover, such high values of *D_xy_* across clades are exceptional, given the expectation that clade-wise comparisons should be scaled downward. These results support the hypothesis that the two divergent CTLMA2 haplotypes arose before the species divergence and have been adaptively maintained as a balanced polymorphism in all three species.

**Table 5:** *D_xy_* among species within and between CTLMA2 haplotype clades and in two nearby loci

*A. coluzzii*	*A. arabiensis**^a^*	*D_xy_*
Clade A	Clade A	0.009
Clade B	Clade B	0.011
Clade A	Clade B	0.034
Clade B	Clade A	0.031
AGAP005540*^a^*	AGAP005540*^a^*	0.016
APL2*^a^*	APL2*^a^*	0.017

*A. gambiae**^a^*	*A. arabiensis**^a^*	*D_xy_*
Clade A	Clade A	0.008
Clade B	Clade B	0.012
Clade A	Clade B	0.035
Clade B	Clade A	0.030
AGAP005540*^a^*	AGAP005540*^a^*	0.017
APL2*^a^*	APL2*^a^*	0.018

a Sequences from published data (Obbard *et al.* 2007).

## Discussion

We examined patterns of genetic variation and divergence in 20 immune-related genes and 17 control genes. We found that, on average, immune genes have elevated rates of amino acid divergence compared to nonimmune genes, and this was particularly true for genes involved in the Imd pathway. Our findings are consistent with studies of other insects, which show that, as a group, immune genes are rapidly evolving (Schlenke and Begun 2003; Sackton *et al.* 2007; Viljakainen *et al.* 2009). The rapid divergence of immune system genes is often hypothesized to be the result of positive selection driving adaptive evolution of the immune system in response to pathogen pressure (Sackton *et al.* 2007). However, when we examined patterns of nucleotide diversity in the genes in our dataset, we found no evidence of recent positive selection driving the evolution of most of the immune genes. For example, although genes in the Imd pathway exhibit elevated amino acid divergence, they also tended to have higher levels of amino acid diversity than the control genes, which is parsimoniously consistent with relaxed purifying selection on this pathway.

Our data are in contrast to those in studies of *Drosophila* and termites that conclude adaptive evolution is particularly common in the Imd pathway. Rapid amino acid evolution has been detected in termites in the terminal NF-κB transcription factor of the Imd pathway (Bulmer and Crozier 2006). In *D. melanogaster*, positively selected sites appear to cluster in the interacting domains of Relish, IKK_β,_ and Dredd, suggesting that the entire Relish cleavage complex is evolving by positive selection (Sackton *et al.* 2007). Likewise, a survey of genes throughout the *D*. *melanogaster* immune system found that the trend of elevated rates of adaptive evolution observed in immune genes overall is primarily driven by genes in the Imd and RNAi pathways (Obbard *et al.* 2009b), although adaptation is not uniform throughout the pathways. It has been hypothesized that pathogen interaction with signaling molecules is driving the rapid evolution of the Imd pathway in *D. melanogaster* (Begun and Whitley 2000). Our findings of relaxed purifying selection in the Imd pathway may seem surprising, because signaling through the Imd transcription factor REL2 has been implicated in antibacterial and antiplasmodium immunity in *A. gambiae*, and given that the Relish cleavage complex is thought to be a site of pathogen-driven evolution in *Drosophila*. However, the specific target or mode of pathogen-driven evolution is likely to be different among insects, shaped by the unique suite of pathogens to which they are exposed, and *A. gambiae* is exposed to a distinct set of pathogens due to its aquatic larval environment as well as its obligate blood feeding and potential exposure to human and animal pathogens. Furthermore, unlike *Drosophila* Relish, the NF-κB transcription factor for the *A. gambiae* Imd pathway, REL2, exists in two isoforms (Meister *et al.* 2005). The full-length isoform (REL2-F) contains an inhibitory domain that must be cleaved off prior to nuclear translocation, whereas the short isoform (REL2-S) that lacks the inhibitory domains is constitutively active (Meister *et al.* 2005). Additionally, some immune responses involving REL2 signaling have been shown to occur independent of the IMD protein (Garver *et al.* 2012), suggesting that novel mechanisms may play a role in REL2 signaling. Our observation of relaxed purifying selection on Imd pathway genes could be consistent with IMD-independent regulation of REL2 signaling in *A. gambiae*.

Evolution in lineage-specific genes or gene families may reflect adaptation to novel pathogens. We found that *STAT-B*, a retrotransposed duplicate of the STAT transcription factor *STAT-A*, which is specific to the Anopheles lineage, shows a strong signature of adaptive evolution, whereas the ancestral copy *STAT-A* is highly conserved. This pattern of selective constraint in *STAT-A* and rapid evolution in *STAT-B* is consistent with a model of neofunctionalization after duplication (Hahn 2009; Lynch and Force 2000). There are seven members of the STAT family in vertebrates, of which different copies have adopted specialized roles in the endocrine or immune system. Interestingly, the vertebrate STAT genes, which are predominantly involved in the endocrine system, evolve more slowly than those that are predominantly involved in immunity (Gorissen *et al.* 2011). The *A. gambiae* JAK-STAT pathway plays a role in regulating nitric oxide synthase in response to bacterial and *Plasmodium* infections, and may additionally regulate TEP1 expression during late-phase *Plasmodium* infections (Gupta *et al.* 2009). A recent study reported that STAT-B, along with various other detoxification and immune genes, is expressed at higher levels in *A. coluzzii* larvae than in *A. gambiae* larvae (Cassone *et al.* 2014), presumably reflecting the shift to the more biotically and abiotically complex larval habitats preferred by *A. coluzzii*, and suggesting that adaptive evolution in *STAT-B* could be important to adaptation to novel ecological or pathogen environments.

Balancing selection can act to maintain functionally important polymorphisms in a population. Genes involved in host–pathogen interactions are considered likely targets of balancing selection, although instances of balancing selection are rarely observed (Leffler *et al.* 2013). We found two distinct haplotype clades in the 5′ end of *CTLMA2*, with 11 fixed SNPs between the clades that result in three amino acid changes near the predicted signal cleavage site. We identified both clades in previously published *A. gambiae* and *A. arabiensis* sequences. Patterns of divergence within and between the *CTLMA2* haplotype clades suggest that they arose before the species divergence and have been adaptively maintained as a balanced polymorphism in all three species, although further study will be required to determine what functional effect the amino acid variants between the two clades might have. CTLMA2 is primarily secreted into the hemolymph in the form of a heterodimer with CTL4 (Schnitger *et al.* 2009); because most of the amino acid substitutions are located in the signal peptide, the different haplotypes might lead to changes in peptide secretion. CTLs are a family of carbohydrate binding proteins that are involved in the immune response through pathogen recognition and as modulators of melanization. The CTL4/CTLMA2 heterodimer is required for successful melanization of Gram-negative bacteria (Schnitger *et al.* 2009), so it is tempting to speculate that the amino acid variants in CTLMA2 might change the configuration of the heterodimer to create a unique carbohydrate recognition profile. In contrast to the protective role against bacterial pathogens, the CTL4/CTLMA2 heterodimer prevents melanization of rodent malaria (Osta *et al.* 2004), suggesting that this heterodimer has conflicting pleiotropic functions in the mosquito immune response that might contribute to the evolutionary pattern observed in *CTLMA2*.

## Conclusion

In this study, we used population genetic analysis to understand how natural selection operates on the *A. coluzzii* immune system. We found evidence of two distinct haplotypes in the *Anopheles*-specific C-type lectin CTLMA2, which have been adaptively maintained as a balanced polymorphism in *A. coluzzii* and the sister species *A. gambiae* and *A. coluzzii*. We also found strong evidence of adaptive evolution in the *Anopheles*-specific JAK-STAT transcription factor STAT-B, consistent with neofunctionalization after duplication. In contrast to these *Anopheles*-specific immune genes, we found no evidence of adaptive evolution in genes involved in the canonical immune signal transduction pathways in *A. coluzzii*. As a group, genes in the Imd pathway exhibit patterns of elevated amino acid diversity and accelerated rates of protein evolution, consistent with relaxed purifying selection, relative to nonimmune control loci. Taken together, these results suggest that host–pathogen interactions involving novel or lineage-specific molecular mechanisms likely play a larger role than canonical immune pathways in adaptively evolving resistance to infection in *A. coluzzii*.

## Supplementary Material

Supporting Information
