# Supplementary material for: Population Genetics of Anopheles coluzzii Immune Pathways and Genes
Source: G3 (Bethesda). 2014 Dec 30;5(3):329–39. doi: 10.1534/g3.114.014845 (PMC4349087; doi:10.1534/g3.114.014845)
Supplement: Supporting Information [file supp_5_3_329__index.html]

Population Genetics of Anopheles coluzzii Immune Pathways and Genes — Supporting Information 

# Population Genetics of *Anopheles coluzzii* Immune Pathways and Genes

## Supporting Information for Rottschaefer *et al.*, 2015

**Files in this Data Supplement:**

- Supporting Information - Figure S1 and Table S1 (PDF, 182 KB)
- Figure S1 - Approximate chromosomal locations of loci sampled. (PDF, 153 KB)
- Table S1 - Population genetic statistics for all sampled loci. (PDF, 96 KB)
