# Supplementary material for: Population Genetics of Anopheles coluzzii Immune Pathways and Genes
Source: G3 (Bethesda). 2014 Dec 30;5(3):329–39. doi: 10.1534/g3.114.014845 (PMC4349087; doi:10.1534/g3.114.014845)
Supplement: Supporting Information [file supp_g3.114.014845_014845SI.pdf]

G3·Genes | Genomes | Genetics

Population Genetics of *Anopheles coluzzii* immune pathways and genes.

Susan M. Rottschaefer*, Jacob E. Crawford[§], Michelle M. Riehle[†], Wamdaogo M. Guelbeogo[‡], Awa Gneme[‡], N'Fale Sagnon[‡], Kenneth D. Vernick[†]**, Brian P. Lazzaro*

*Department of Entomology, Cornell University, Ithaca, NY 14853, [§]Department of Integrative Biology, University of California, Berkeley, Berkeley, CA 94720, [†]Department of Microbiology, University of Minnesota, St. Paul, MN 55108, [‡]Centre National de Recherche et de Formation sur le Paludisme, 01 BP 2208 Ouagadougou, Burkina Faso, **Unit of Insect Vector Genetics and Genomics, Institut Pasteur, Paris, France.

Corresponding Author: Susan M. Rottschaefer , Department of Entomology, Cornell University, Ithaca, NY 14853. E-mail: smr53@cornell.edu

DOI: 10.1534/g3.114.014845

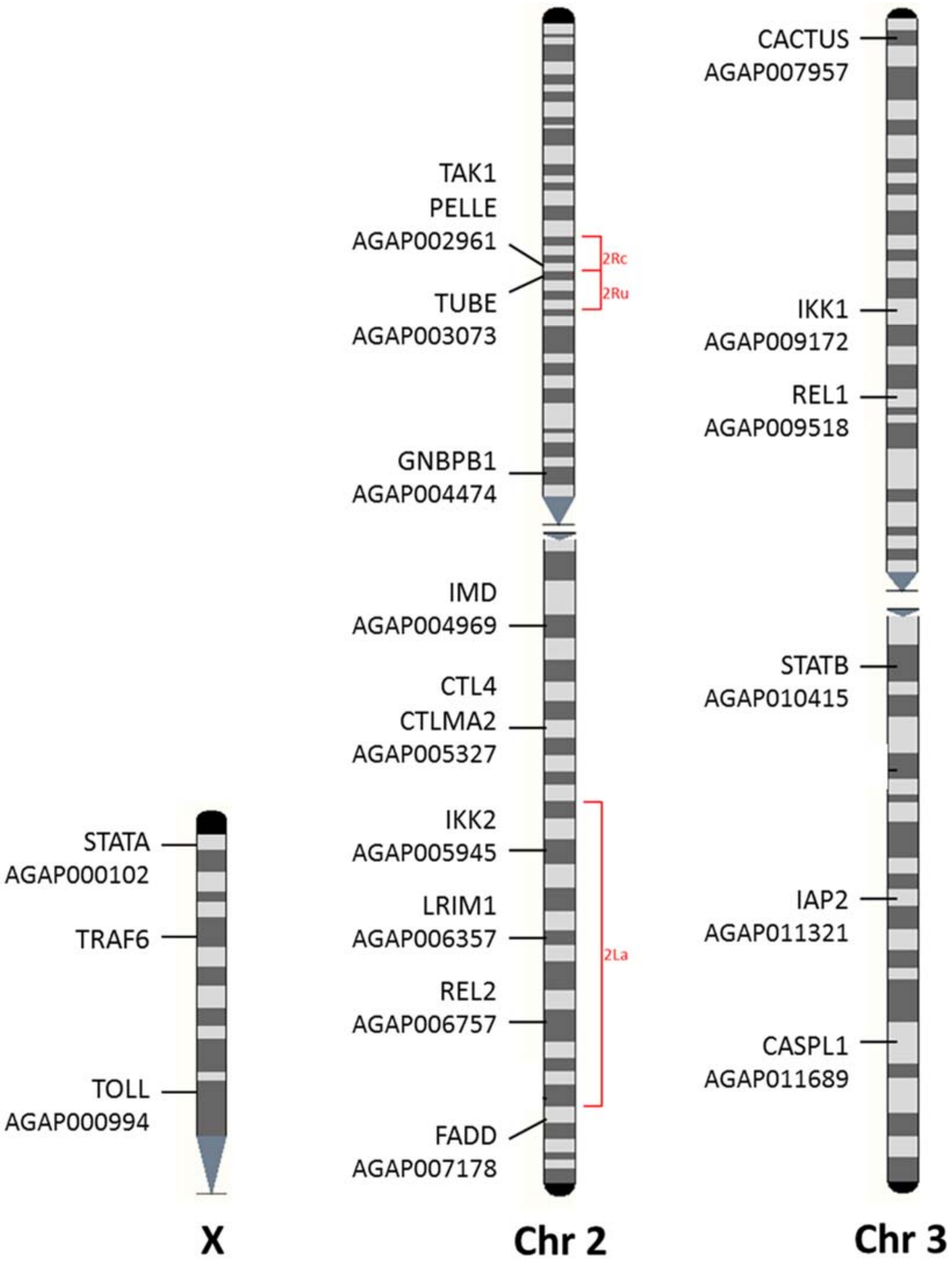

Figure S1 Approximate chromosomal locations of loci sampled. AGAP identifiers are provided for all non-immune control loci. Control loci are located within 40-100KB of their "matched" controls. Positions of chromosomal inversions are shown in red.

Table S1 Population genetic statistics for all sampled loci

locus	N^a	Sites^b	S^c	π_{total}^d	π_s^e	π_a^f	D^g	K_{total}^h	K_S^i	K_A^j	K_A/K_S^k
CACTUS	20	2660	133	0.0132	0.0215	0.0021	-0.201	0.0247	0.047	0.0016	0.033
CASPL1	19	1424	133	0.024	0.0608	0.0116	-0.385	0.0269	0.061	0.0134	0.212
CTL4	20	882	44	0.0125	0.0311	0.0039	-0.263	0.0326	0.06	0.0147	0.237
CTLMA2	20	891	55	0.0217	0.0258	0.0084	1.254	0.047	0.1021	0.0204	0.188
FADD	20	980	124	0.0294	0.0458	0.0149	-0.601	0.0447	0.0663	0.0248	0.363
GNBPB1	20	1782	79	0.0102	0.0202	0.0013	-0.697	0.0334	0.0773	0.0064	0.078
IAP2	20	873	78	0.0253	0.0451	0.006	0.3	0.0467	0.0783	0.0131	0.16
IKK1	20	2681	170	0.0141	0.0336	0.0051	-0.987	0.0294	0.0728	0.0077	0.102
IKK2	20	2035	98	0.0119	0.0373	0.0043	0.116	0.037	0.0795	0.0082	0.098
IMD	20	1270	65	0.0116	0.0195	0.005	-0.804	0.0328	0.0481	0.0215	0.439
LRIM1	20	1636	76	0.0106	0.0265	0.0055	-0.892	0.0302	0.0638	0.0179	0.272
PELLE	20	1986	87	0.013	0.0325	0.0039	0.261	0.0342	0.0751	0.0096	0.125
REL1	19	6802	363	0.016	0.0226	0.0024	0.238	0.03	0.0493	0.0055	0.108
REL2	19	5075	311	0.013	0.0288	0.0038	-0.912	0.0151	0.0338	0.004	0.116
STAT-A	20	2740	55	0.0042	0.0064	0	-1.111	0.0221	0.0268	0	0
STAT-B	19	2123	15	0.0018	0.0021	0.0015	-1.025	0.0377	0.0627	0.0295	0.46
TAK1	20	5409	395	0.0163	0.037	0.0023	-0.819	0.0359	0.0506	0.0044	0.084
TOLL	20	3595	40	0.0017	0.0054	0.0004	-1.802	0.0225	0.066	0.0065	0.094
TRAF6	20	1814	53	0.0048	0.0079	0.0023	-1.713	0.0574	0.1132	0.0181	0.149
TUBE	18	2285	110	0.0156	0.0365	0.0087	0.081	0.0305	0.0711	0.0131	0.176
AGAP000102	20	3500	131	0.0078	0.0104	0.0034	-1.218	0.0498	0.0388	0.0145	0.368
AGAP000994	20	2519	47	0.0027	0.0049	0.0001	-1.98	0.0288	0.0477	0.0019	0.039
AGAP002961	20	4700	237	0.0116	0.0315	0.0017	-0.58	0.0208	0.0504	0.0032	0.062
AGAP003073	20	1768	115	0.0152	0.0241	0.0003	-0.743	0.0393	0.0895	0.0033	0.035
AGAP004474	19	2363	150	0.0181	0.0282	0.0013	-0.077	0.0442	0.0579	0.0031	0.052
AGAP004969	20	1653	64	0.009	0.0209	0.0034	-0.753	0.022	0.0594	0.0053	0.086
AGAP005327	19	1088	54	0.0102	0.0208	0.0007	-1.107	0.0099	0.0117	0.0003	0.029
AGAP005945	19	2553	183	0.0169	0.0367	0.0016	-0.678	0.0487	0.0562	0.0027	0.046
AGAP006357	20	3166	213	0.0183	0.0336	0.0013	-0.086	0.034	0.0728	0.0065	0.085
AGAP006757	20	5014	409	0.0183	0.0204	0.0004	-0.868	0.0489	0.0406	0.0002	0.005
AGAP007178	20	1699	84	0.0105	0.0253	0.0017	-1.017	0.0203	0.0424	0.0032	0.074
AGAP007957	20	4364	317	0.0158	0.0293	0.0029	-0.915	0.0287	0.0666	0.0065	0.094
AGAP009172	19	2826	125	0.0115	0.0242	0.0006	-0.4	0.0269	0.0492	0.0021	0.042
AGAP009518	19	4695	411	0.021	0.0253	0.0021	-0.709	0.039	0.0267	0.0014	0.051
AGAP010415	20	1275	25	0.0035	0.0117	0.0011	-1.188	0.0271	0.0947	0.0021	0.02
AGAP011321	20	1566	89	0.0142	0.0376	0.005	-0.381	0.0318	0.0809	0.0134	0.158
AGAP011689	20	1684	96	0.0153	0.041	0.0003	-0.132	0.022	0.0414	0.0029	0.067

[a]Number of *A. coluzzii* alleles sampled
[b]Number of base pairs sequenced per allele
[c]Number of segregating sites
[d]Pairwise genetic diversity calculated for all sites
[e]Pairwise genetic diversity calculated for synonymous sites

[f]Pairwise genetic diversity calculated for nonsynonymous sites
[g]Tajima's D calculated using silent sites.
[h]Pairwise divergence at all sites between *A. coluzzii* and *A. merus*
[i]Pairwise silent divergence between *A. coluzzii* and *A. merus*
[j]Pairwise replacement divergence between *A. coluzzii* and *A. merus*
[k]K_A/K_S ratio between *A. coluzzii* and *A. merus*
