# Supplementary material for: Population Genetics of Anopheles coluzzii Immune Pathways and Genes
Source: G3 (Bethesda). 2014 Dec 30;5(3):329–39. doi: 10.1534/g3.114.014845 (PMC4349087; doi:10.1534/g3.114.014845)
Supplement: Supporting Information [file supp_g3.114.014845_FigureS1.pdf]

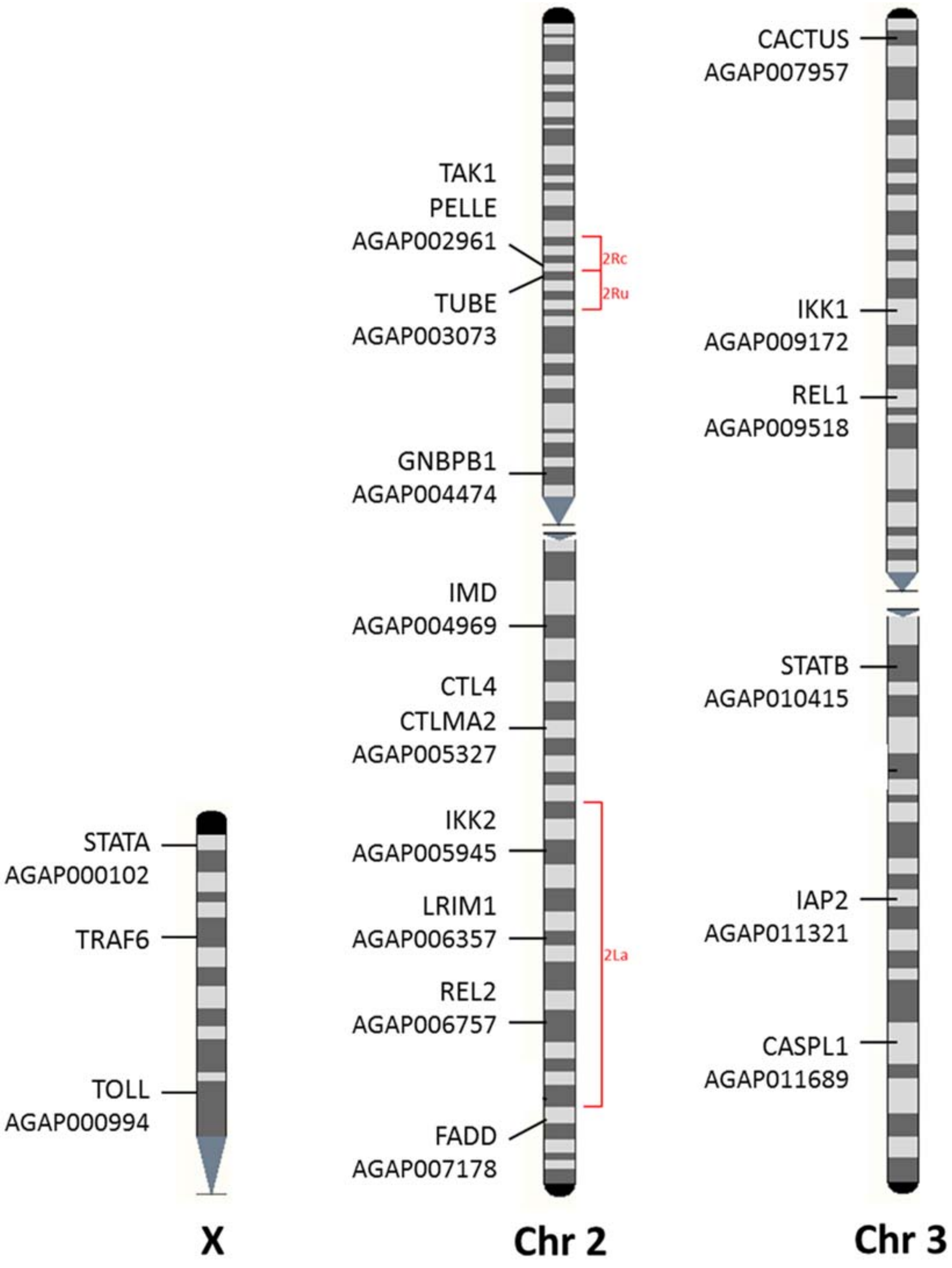

Figure S1 Approximate chromosomal locations of loci sampled. AGAP identifiers are provided for all non-immune control loci. Control loci are located within 40-100KB of their "matched" controls. Positions of chromosomal inversions are shown in red.
